# Preparedness for Caregiving Role and Telehealth Use to Provide Informal Palliative Home Care in Portugal: A Qualitative Study

**DOI:** 10.3390/healthcare12191915

**Published:** 2024-09-25

**Authors:** Paula Caetano, Ana Querido, Carlos Laranjeira

**Affiliations:** 1 School of Health Sciences, Polytechnic University of Leiria, Morro do Lena, Campus 2, Alto do Vieiro, Apartado 4137, 2411-901 Leiria, Portugal; pcaetano67@hotmail.com (P.C.); ana.querido@ipleiria.pt (A.Q.); 2 Centro de Saúde de Ourém, Unidade Local de Saúde da Região de Leiria, Rua Dr. Armando Henrique dos Reis Vieira, 2490-546 Ourém, Portugal; 3 Centre for Innovative Care and Health Technology (ciTechCare), Polytechnic University of Leiria, Campus 5, Rua das Olhalvas, 2414-016 Leiria, Portugal; 4 Center for Health Technology and Services Research (CINTESIS), NursID, University of Porto, 4200-450 Porto, Portugal; 5 Comprehensive Health Research Centre (CHRC), University of Évora, 7000-801 Évora, Portugal

**Keywords:** caregivers, home care, palliative care, preparedness, telehealth, qualitative study, Portugal

## Abstract

**Background/Objectives:** Given the increasing occurrence of long-term illnesses, it is imperative to focus on adequately preparing and assisting those who assume the responsibility of caregiving. Our study aims to explore whether caregivers feel prepared to provide informal palliative home care, their experiences, and the usefulness of telehealth in managing daily activities. **Methods:** Using a descriptive qualitative research design and a purposeful sampling technique, thirteen primary family caregivers who provide informal palliative home care were recruited. Data collection was conducted through face-to-face individual interviews conducted from May 2023 to July 2023. Data were analyzed using Braun and Clarke’s reflexive thematic analysis. **Results:** Caregivers were mainly female (n = 8) with a mean age of 59.5 years (SD = 9.42). Based on our findings, three overarching themes emerged: (1) becoming a caregiver, (2) support-from-home palliative care team, and (3) telehealth in palliative home care. The reasons that influence the preparedness of family caregivers include their own desires, health conditions, their range of responsibilities, and the consequences that arise from the situation’s complexity. Telehealth helps fulfill the patient’s wishes to be at home in EoL and provides caregivers with access to professional guidance and support. **Conclusions:** Specialized home-based palliative care teams must be aware of caregivers’ self-assurance, knowledge, skills, and aptitudes in carrying out daily responsibilities and in managing emotions to improve preparedness for caregiving, loss, and its aftermath. The provision of professional PC services in the home along with a robust support system for informal caregivers is invaluable.

## 1. Introduction

Palliative care (PC) plays a crucial role in enhancing the quality of life, particularly during the later stages of illness, and aims to alleviate the burden of the condition. The World Health Organization (WHO) has provided a definition of PC as a strategy that enhances the quality of life for patients and their families who are facing challenges associated with life-threatening illnesses [1]. It is delivered through the management, evaluation, and therapy of pain and other bodily, psychological, and spiritual issues. Since the release of the Quality of Death Index in 2021 [2], there has been a growing emphasis on enhancing the comfort and autonomy that individuals experience during their final stages of life. As highlighted in the Quality of Death Index, end-of-life (EoL) care preferences serve as an indicator of the standard of care provided by PC [2]. While several individual, structural, and contextual factors influence the place of death, a recent umbrella review found that most people preferred to die at home [3]. The crucial factor enabling death at home is the presence of family members or close individuals and their preparedness to assume the responsibility of delivering EoL care for those who desire to die in their own homes [4]. In the context of PC, the term “family” encompasses not just formal relationships but also those that are self-defined or identified by the patient as significant. In this vein, family caregivers (referred to henceforth as caregivers) include family members, friends, or other people who have emotional and social bonds with a patient. They are unpaid lay people with a close supportive role who share the patient’s illness experience and who undertake vital care work and emotional management [5]. However, caregivers often lack adequate training to effectively carry out caring responsibilities, especially during the transition from hospital to home [6,7,8]. Consequently, caregivers frequently experience a sense of unpreparedness when it comes to caring for a person with palliative needs residing at home [9,10,11]. Several challenges were found during PC transitions from acute care to community-based care, including physical discomfort, medication-related disorientation, ambiguity regarding healthcare obligations, and emotional turmoil [12]. A lack of caregiver preparedness may lead to unforeseen hospital admissions, resulting in significant financial burdens on the healthcare system, potential health hazards for patients, and an augmented workload for caregivers [13]. Caregivers derive purpose from their responsibilities, which motivate them to effectively manage the difficulties associated with taking care of an individual in need [14]. To alleviate the burdens of caregiving and facilitate the ability to maintain a purposeful life both within and outside of their caregiving responsibilities, it is necessary to provide supplementary support [14,15]. Studies have found several important factors related to receiving PC at home, including healthcare accessibility, caregiver support, tailored person-centered care, multidisciplinary care provision, and quality improvement [16,17].

Several studies have demonstrated that specialized home PC teams who provide support for patients and their families at home can result in a higher proportion of individuals being able to die in their own homes, thereby reducing hospital admissions and hospital stays [18,19,20]. The model of PC organization in Portugal [21] includes palliative care units that offer in-patient care; Hospital Support Palliative Care Teams that provide PC advice and support to the entire hospital structure; and finally, Home Palliative Care Teams (HPCT) that care for patients in their homes, support their families and caregivers, and advise family doctors and nurses who provide care at home [21]. The model implemented in Portugal exemplifies the principles of accessibility and fairness. However, a need for expansion has become apparent, especially in the realm of home-based palliative intervention. Due to financial and structural barriers, as well as low political priority, there are inequalities in access to HPCT [22]. There is evidence of a lack of awareness and comprehension of PC among the Portuguese general population [23]. More exactly, there are misconceptions regarding the goals of palliative care that delay timely PC referrals and restrict informed decision-making.

Given the increasing occurrence of long-term illnesses, it is imperative to focus on adequately preparing and assisting persons who take on the responsibility of caregiving [24]. Caregiving has been linked to diverse outcomes, both positive (like personal development) and negative (like emotional fatigue) [25]. Prior studies have identified that those assuming the responsibility of a caretaker are more susceptible to experiencing injuries and illnesses, including anxiety and despair [26,27]. Some persons may experience caregiver burden due to the growing and intricate nature of some chronic diseases. Specifically, the shift to a demanding caregiving position, where individuals assist with the activities of daily living, was discovered to be linked to the deterioration of caregiver functionality [28]. Individuals may encounter varying levels of burden depending on the nature and regularity of their caregiving responsibilities, as well as their attitudes towards the activities and challenges associated with providing care.

A person-centered approach that prioritizes the intricate needs of the individual rather than the physical aspects of care has not yet been universally adopted in PC settings [29]. Evidence also revealed deficiencies in inter-professional communication, fragmentation of care, and coordination of care [30]. These challenges can be mitigated through digital health technology (DHT) using different telecommunication tools (i.e., a smartphone, tablet, or desktop) to provide healthcare remotely in an interactive or passive mode that could benefit caregivers and care recipients at home. For instance, evidence demonstrates the beneficial effects on caregivers’ self-esteem, self-efficacy, mastery, and burden [31]. Given that various chronic ailments can provide distinct difficulties for caregivers, many studies have specifically examined individual chronic illnesses, such as dementia [32] or cancer [33]. Support in various domains may be required by caregivers, and the customization of digital devices has been proposed as a means to address individual caring needs [34]. Specifically, tailoring digital devices and promoting digital literacy could aid in addressing concerns related to the volume and accuracy of general health data, such as excessive information and subpar readability [35,36].

Although earlier studies have examined a wide range of DHT, there is a lack of literature on carers’ perceptions of the usage of telehealth to assist in daily activity management [37]. Regarding caregivers who play a major role in caring for people with PC needs, studies that use a qualitative research approach and focus on their experiences are still scarce in the Portuguese context. Therefore, this study aims to explore how caregivers feel prepared to provide informal palliative home care, their experiences, and the usefulness of telehealth in managing daily activities. We hope to contribute a better understanding of their importance for caregivers wishing to support death at home.

## 2. Materials and Methods

### 2.1. Study Design

This qualitative descriptive study is part of a wider research project that aims “to co-design, develop, and test the feasibility of the Help2Care-PAL mHealth app that addresses the needs of caregivers of palliative patients cared for at home” [38]. Qualitative methodologies are valuable for examining the attitudes, values, and motivations that underlie individual decisions on health [39]. In this vein, the social constructivism theory underpins this study, and knowledge was co-created through the interaction between researchers and participants [39].

This study adheres to the consolidated standards for reporting qualitative research (COREQ) recommendations [40] (Supplementary Table S1).

### 2.2. Sample and Recruitment

Using a purposeful sampling technique [41], caregivers were invited to take part in this study if they fulfilled the following inclusion criteria: (a) Men or women aged at least 18 years with close bonds to the person receiving PC at home, i.e., based on the NECPAL tool; patients were included if they positively answered the question “*Would you be surprised if this patient dies in the next year?*” and if they also fulfilled at least one of clinical indicators in terms of disease severity and progression and the disease-specific indicators [42]. (b) Unpaid primary carers who provide informal palliative home care. (c) Primary carers residing with the patient or living near the patient. Participants were excluded if they provided care to family members residing in nursing homes, if they had cognitive impairment, or if they could not understand and speak Portuguese.

To determine the number of participants for this study, we used the criterion of data saturation. Recruitment stopped when including more people or data no longer contributed important information [43].

### 2.3. Data Collection

Data collection was conducted through face-to-face individual interviews conducted from May 2023 to July 2023. Suitable caregivers were preselected by the staff of a HPCT from the Alentejo region (Portugal). Subsequently, the first author (P.C.) contacted participants via telephone, providing them with detailed information about the study and scheduling initial interviews, including the time and location. Every caregiver supplied written consent after being fully briefed. The interviews were conducted in a private room within the participant’s home. The risk of being perceived as intrusive was balanced against the benefits of establishing a safe environment and demonstrating sensitivity to requirements.

The semi-structured interview guide drew from the current research [38,44,45] and the authors’ clinical expertise in the subject. It included the following topics: (a) perceived preparedness for different domains of caregiving, including communication with relatives and considerations about the caregivers’ concerns; (b) caregivers’ perspectives and challenges when caring at home and using telehealth in health-seeking behaviors.

We conducted a pilot interview and made some minor adjustments, which allowed for the incorporation of the pilot interview as part of our data. The first author (P.C.) carried out all individual face-to-face interviews. Interviews lasted between 40 and 60 min and were audio-recorded and transcribed verbatim. Each interview commenced as an open conversation when caregivers were encouraged to express their experiences and preferences regarding their role as caregivers and their requirements for home care support. The interview guide served as a prompt during the interview to guarantee that the aforementioned subjects were addressed. Open questions, such as inquiries about individuals, objects, or concepts, were posed to expand or refine the scope of interest. Interview notes were made after each interview, mainly to describe the atmosphere during the interviews. No repeat interviews were carried out. Further, all transcripts were returned to participants for comments and/or corrections.

Given that the interviews were conducted in European Portuguese, the interview extracts were translated and subsequently back-translated to ensure the preservation of the original meaning. Alpha-numeric codes (P1, P2, …) were associated with data extracts to safeguard the anonymity of caregivers.

### 2.4. Data Analysis

Inspired by Braun and Clarke’s reflexive thematic analysis, data were analyzed to define the conceptual framework of the main topics that emerged from the interviews [46]. Table 1 depicts the six-phase process as proposed by Braun and Clarke [47]. Every interview was carefully analyzed, and the process of coding commenced using phrases that were the length of a sentence from the text. The codes were categorized into themes and modified following each interview. After completing five interviews, the coding process transitioned to including lengthier phrases consisting of multiple sentences. An attempt was made to transition from descriptive codes to interpretive codes in order to discern the wider relationships among participants’ experiences. Following the completion of each interview, the coding matrix underwent a thorough evaluation to identify the prominent elements in the participants’ experiences [46]. Qualitative data analysis software WebQDA (Version 3.0, Universidade de Aveiro, Aveiro, Portugal) was used to facilitate data storage and management.

The research team used an inductive approach to generate thematic categories. Several research meetings were conducted to facilitate the sharing and comparison of the findings, as well as the identification of common themes. If there were differing perspectives, a decision was made by consensus. All researchers included in this study are registered nurses (RN) with diverse perspectives and firsthand experiences in the field of EoL care. The main researcher (P.C.) is a female mental health nurse specialist who works in community care and has practical experience with PC individuals. The second and third researchers possess expertise in working, teaching, and researching in the field of PC, as well as expertise in qualitative studies (A.Q. and C.L.). All researchers have experience as informal caregivers of their older loved ones and faced the difficulties of balancing their professional, familial, and caregiving roles. These diverse experiences enhanced the interpretation of the material and decreased potential biases.

### 2.5. Trustworthiness

We employed Guba and Lincoln’s trustworthiness criteria [48]. Firstly, we established credibility by employing investigator triangulation and collecting qualitative data from multiple sources, such as interview transcripts, quotations, and researcher notes. Keeping an open-minded attitude, recognizing personal biases, and practicing self-reflection throughout the entire study process helped validate findings and enhanced their credibility. Secondly, we achieved transferability by providing comprehensive descriptions of the study design, participants, context, sampling, data collection, and analysis. Thirdly, we ensured dependability by subjecting our study to an external audit conducted by other researchers. Lastly, we attained confirmability by documenting reflexive reports during data collection and analysis. Peer debriefing and reflexive journaling enabled researchers to record their thoughts, biases, and reflections, promoting transparency and reducing subjectivity [48].

### 2.6. Ethics

This study was carried out in compliance with the Declaration of Helsinki and the European General Data Protection Regulation (GDPR2016/679). Permission was sought and granted by the Ethics Committee of Unidade Local de Saúde—Baixo Alentejo (code: 7.4.1. approved on 15 February 2023). Before harvesting data, participants were informed about this study’s objectives and the possibility of terminating their participation without any consequences. Those who agreed to participate signed an informed consent form. There were no financial rewards associated with participation. The personal data of the participants was anonymized in the material, and only the authors had access to it. All participant data were compiled in a digital folder only accessible by the principal investigator. Archives are to be destroyed one year after completion of the study.

Participants were provided with follow-up contacts if they experienced distressing emotions or thoughts during the interviews. A follow-up contact was not requested by any of the participants.

## 3. Results

### 3.1. Sample Characteristics

In total, 13 individual interviews with family carers were conducted. Eight were female with a mean age of 59.5 ± 9.42 years (range of 44 to 82 years). The main participants in this study were primarily spouses (n = 5) or children (n = 7) who were responsible for providing care to their family members. The time in the caregiving role varied between 4 months and 15 years (average time = 40.5 months). Most care recipients had a cancer illness trajectory (n = 8). A sample description is provided in Table 2.

### 3.2. Overview of Findings

The thematic analysis resulted in three themes and seven subthemes, as indicated in Table 3. The factors that influence the preparedness of caregivers include their own desires, health conditions, their range of responsibilities, and the consequences arising from the situation’s complexity. Furthermore, telehealth helped caregivers in their daily activities at home, namely in symptom management and self-care promotion. However, participants indicated some drawbacks and challenges. These findings are discussed in more detail in the following sections.

#### 3.2.1. Became a Caregiver

This main category outlines the nature of becoming a caregiver either by personal choice or imposed circumstances.

(a) Caregiving as a natural decision

Participants demonstrated that they became caregivers by voluntary choice, either by their own decision or the influence of others. This was driven by cultural imperatives, leading to a strong desire to help as much as possible. P3’s switch to caring was motivated by her desire to reciprocate the attention her father had provided her during his youth: *I don’t want to give up my father, I don’t see being at home or with him longer as a sacrifice. In fact, I take advantage of every moment I can to be with him.*

Similarly, P8’s choice to engage in caregiving was motivated by a strong sense of filial reciprocity towards her mother:

*I chose to be a caregiver. I do this with a lot of love and it was always something I wanted to do. It was taking care of my mother, because she was a caregiver and I absolutely didn’t want anyone else to take care of her.*

Although family caregivers had no prior experience of caring for someone with PC needs, they expressed a desire to uphold the patient’s overall health and comfort by enabling EoL care in a setting that was both comfortable and familiar to the patient.

P7: *I had never taken care of anyone, and I needed to learn something. Even though it’s different from what a physiotherapist does, I know that when she needs it and he can’t, I can help her in some way with the exercises so that she feels more comfortable.*

P5: *Yes, it’s not only a benefit for me, but I think it’s also a benefit for my mother. I always try to make sure she stays “alive”, connected with us, even though sometimes that’s difficult.*

Two participants also expressed their intention to safeguard and assist the patient, which influenced the families’ choice to manage EoL care in their own home.

P3: *Being focused essentially on the person’s well-being or on transmitting tranquillity, love, and security to the people we are caring for, I think is fundamental.*

Pl3: *I feel good about complying with his wishes. He’s fine, after all he’s home, in the place he always wanted to be.*

(b) Imposed caregiving

Some participants experienced a change in their circumstances that was not within their power or choice but was influenced by social expectations.

P1: *We are three brothers and none of them showed interest in taking care of my mother. I ended up having to take over (…) I stopped having a life of my own, I had to give up a lot of things.*

P6: *I am married to her, and I have responsibilities towards my wife. I had no other option.*

Additionally, handling multiple roles and a feeling of duty to provide care and support also hindered the desire for marital independence.

P8: *Today, I recognize that the need for permanent care made me put my marriage on the back burner. My husband and I have given up several things on several occasions so that I can always be present.*

Being a caregiver intersects with different areas of life. Caregivers face difficulties when handling conflicting demands between caregiving and other important duties and obligations, which are described as “overwhelming” and “disruptive”. Caregivers also grappled with caregiving demands imposed by others when sometimes they felt limited due health constraints.

P9: *With dementia, he (*husband*) sometimes becomes aggressive, which makes me feel extremely overwhelmed. I try not to value it, but it’s very difficult. My daughter tells me to go ahead, because he is very sick. But it hurts to hear what he says, because I also have my health problems and I try very hard to give him the best.*

P12: *Despite trying to always be there to support my mother at this stage, I have to maintain another professional activity to cover expenses, which is very demanding.*

#### 3.2.2. Support from Home Palliative Care Team

This theme reflects the person-centered care promoted by the PC team, whether through close communication or by supporting the management of complex situations and shared decision-making.

(a) Compassionate caring relationships

Caregivers believed that these interactions enhanced PC professionals’ comprehension of their caregiving requirements and that a strong rapport with professionals fostered caregivers’ sense of being appreciated as individuals and, to some degree, relieved feelings of isolation and disbelief.

P1: *I can’t imagine what my mother would have become if it weren’t for them (the team). We live in a remote village and contact with the team helped reduce the isolation we felt. She often has crises and whenever I need to, I contact the team who helps me deal with the uncontrolled symptoms.*

Despite P4’s initial negative stereotype that associated PC with abandonment, over time, she reported having changed her perception.

P4: *I always thought that palliative care was used when there was nothing else to do. My daughter worked in palliative care and helped me change my mind. Furthermore, the community palliative care team has been a fundamental anchor in monitoring my mother. They are very kind, and we have built a relationship of trust.*

(b) Managing complex situations and emotional turmoil

Most of the participants expressed significant concerns over intense anxiety and worry associated with the uncertain fate of the patient’s decline towards the EoL. They emphasized how this impacted their overall well-being. Further, participants highlighted the emotional challenge when they thought about impending death and future life.

P4: *The biggest concern is having to watch him suffer (father) and trying to understand what he is feeling. The anguish that is generated is the worst thing for those who care (…) When I see him worse, I think that death is near, and it makes me feel so sad.*

P2: *My concern is facing existential suffering and the human frailty when death is near. I talk openly about imminent death with the team, but it’s so difficult though to talk about loss and its aftermath. What will it be like afterwards?*

Families must deal with abrupt fluctuations in the patient’s well-being without sufficient understanding of what to anticipate and how to respond. Consequently, it is unsurprising that caregivers (as well as patients) experience distressing situations intertwined with the patient’s emotional connection to their family. The complexity of daily care situations generates in caregivers a “merry-go-round of emotions”.

P11: *It’s a mix of feelings. When there is a good day, I can get up and be filled with joy. But, on the other hand, when things get worse, there is sadness and frustration. Yesterday I cried with emotion when I saw him sitting. But then there’s always a brake, something that doesn’t let me live to the fullest… But the palliative care team helps me deal with these challenges.*

P7: *When they come to the house, they are never there to clock in or do chores, everything they do is with love and care. Besides, they are always available. I have had to contact nurses by phone, and even if they don’t answer right away, they return the call as soon as possible. For us caregivers, this is a comfort, especially when we are having a lot of difficulties. I give you the example of fan leaks (i.e., BIPAP) which sometimes leave me distressed.*

Occasionally, there were instances of discord between the desires of patients, the capabilities of caregivers, and the perception of what was appropriate for the patient. This could impact the standard of care delivered and the overall carer experience. The HPCT assists in resolving care conflicts, particularly when patients’ nourishment or eating declines just a little.

P12: *What we desire is not always the same as what others value. I realized this when my mother asked me to leave her in bed and I insisted on getting her up. After all, what I wanted was for her to get better. But my expectation was unrealistic. The nurse told me that I was doing a good job, after all I just wanted to do my best…*

P6: *In addition to her physical limitations, the most difficult thing is being able to feed her. I’ve been told not to worry, but it’s so hard to watch her waste away.*

Finally, one participant stated it was through the HPCT that he became aware of the existence of peer-support groups for caregivers that offer the possibility to openly discuss challenging events and gain insight into how others manage them.

P7: *The team doctor told me about the APELA (Portuguese Association of Patients with Multiple Sclerosis) which offers support to caregivers. It was from there that I met other people experiencing identical processes, which is very important for exchanging experiences. After all, we are “not alone” in our experiences and emotions.*

(c) Participating in treatment decision-making

All participants expressed complete satisfaction with the services provided by HPCT and appeared mainly at ease in delivering patient care in a home setting. Many participants were inspired by a PC team in terms of their comprehension of palliation and the standard of caregiving. They expressed a desire for the patient to avoid being admitted to the hospital.

P7: *There were several times when I was heard by the team about my wife’s condition, the objective was to keep her comfortable, avoiding a trip to the emergency department.*

P2: *Our goal as a family has always been to ensure the best for my grandmother, and when we were told that we could care for her at home with the support of the team, that was very important. It was the care we wanted, and our wishes were respected.*

Providing instruction to caregivers on medical interventions, such as administering subcutaneous injections and treating wounds, as well as ensuring that professionals are available around the clock for medical guidance, was deemed very important by caregivers. Receiving counseling for EoL and emotional support was also found to be beneficial.

P6: *I have the support of the team…. Her tumour was external and was bleeding a lot. She even had radiotherapy and the wound was very “ugly”. It wasn’t easy for me to treat, but I did it based on the guidance the team gave.*

P4: *When my mother had severe pain and would not give in to oral morphine, they switched the opioid to the subcutaneous route and taught me how to manage the therapy. At first it wasn’t easy, but when I realized that she got better it made me feel valued.*

P5: *In moments when I am less well, the team understands this and tries to help me with guidance. They are always available when I need. I feel listened to, which is very important when we live in constant stress… sometimes we just need to be heard and have our concerns attended to.*

Interestingly, one participant reinforced the role of the caregiver in detecting changes in patients’ behavior that are sometimes underestimated by professionals.

P4: *The fact that you are with her 24 h a day, 7 days, makes you more sensitive to identifying changes that when the nurses come to the house, they cannot perceive. So, I try to talk to them and participate in decisions about adjusting therapy.*

#### 3.2.3. Telehealth in Palliative Home Care

Although there are limitations to telehealth use, this theme underlines that its use enhances and expands caregivers’ ability to connect with PC professionals from the comfort of their homes.

(a) Symptom management and self-care promotion

Caregivers considered that telehealth provided them with more access to professionals in emergency situations (i.e., whenever they required immediate assistance), giving them a feeling of not being alone. They experienced a sense of relief during emergency situations when the staff would send them a text message containing the name of the medication to be administered, and thereafter provide a follow-up either on the same day or the next day.

P3: *Not long ago he (father) had a crisis bout of hiccups, and it was exasperating… and I asked the team to do a teleconsultation, which helped me understand what I could do immediately. They also sent me messages with information that supported care.*

The caregivers needed to be aware that professionals were accessible to them, providing care and monitoring them through telehealth. This fostered emotions of nurturance, attachment, alleviation, serenity, and heightened safety.

P5: *I have never used digital resources but knowing that they are available generates security and some relief.*

Monitoring symptoms at home allowed for the effective reporting of symptoms to the HPCT, and conducting follow-ups remotely was seen as less intrusive compared to a phone call. Participants in teleconsultations with HPCT experienced focused responsiveness and opportunities for direct consensus on the allocation of responsibilities for future actions.

P1: *I make a point of recording the frequency and intensity of symptoms, I use the Edmonton scale, and then I send it via WhatsApp to the team and they tell me what I should do.*

When PC needed to be increased, participants mentioned that telehealth may intensify face-to-face support as physical function worsens. P2 stated: *Telehealth enables me to discern those who necessitate additional in-person assistance. In my situation, as my husband’s health continues to decline, professionals visit him more regularly since he is no longer able to utilize telehealth systems.*

Lastly, P10 mentioned that the professionals on the team recommended a free app with some mindfulness exercises to relax: *Since I started using it, I have felt greater peace of mind, I never thought it would work, but I feel good. These are moments of self-care that are very important for us caregivers*.

(b) Drawbacks and challenges of telehealth

However, some caregivers had a sense of insecurity when utilizing telehealth due to their limited digital literacy. While participants recognized the value of telehealth, limited knowledge, unfamiliarity with the technology, and misinformation posed barriers to the utilization of telehealth.

P3: *Digital information has very good things, but we have to know how to filter… the information is not always the most appropriate.*

P6: *I have difficulty using cell phones… they offered me a smartphone and I had to give it up because I couldn’t get used to it and I always had to get help from my grandchildren. I’m from the time when these things didn’t exist. They’ve already explained it to me, but I have difficulty handling it.*

## 4. Discussion

To the best of our knowledge, this is one of the first attempts to explore caregivers’ preparedness to provide informal palliative home care, their experiences, and how helpful telehealth is to managing daily activities. The knowledge gained from this study is particularly intriguing as it provides a more profound comprehension of home-based PC interventions and their potential impact on caregivers.

Several reasons influence family members to offer home-based PC for their ill relatives. Among these factors, family values were identified as a significant driving force leading a family member to decide to provide care and assume the role of caregiver [45,49]. Family caregivers aimed to preserve the patient’s well-being by enabling EoL care in a location the patient would perceive as pleasant and familiar [11,45,49,50]. Adult children who had cared for an ailing parent felt a sense of obligation to reciprocate the affection they had received from their parents, which was also a significant influence [51,52]. Despite challenging circumstances, a family’s determination to safeguard and assist the patient formed the foundation for their choice to administer EoL care in the comfort of their own home [52]. The combination of these values enabled family members to maintain their dedication to their caregiving responsibilities and provided the necessary strength to navigate the intricate difficulties associated with caregiving [49]. As underlined by the evidence, the concept of reciprocity of care refers to the caregivers’ sense of giving back to their loved ones [53]. This is strongly influenced by cultural and religious beliefs, as well as the assumption that family caregivers should receive assistance.

The findings emphasize that most caregivers had no prior experience in providing care for people with PC needs, which aligns with previous studies indicating that caregivers can acquire expertise and improve their skills over time through hands-on caring experiences or by studying the caregiving practices of others [54]. Our findings also suggest that some family members experienced a sense of obligation to assume the role of caregiver and a limited sense of autonomy due to societal norms that expect them to provide care for an ill family member [52]. Family members who possessed a strong inclination to safeguard the patient reported feeling more prepared for their position, but those who were affected by societal standards often expressed feelings of unease. Managing various duties can result in excessive strain, and it is crucial to maintain a balance between these conflicting responsibilities [55]. In our study, the importance of maintaining a balance was particularly notable, particularly when the responsibilities of being a caregiver were overshadowed by other familial obligations.

A significant number of caregivers reported obtaining assistance from PC services. Implementing home-based PC allowed family caregivers to feel better equipped and ready for the challenges ahead [45,49]. In practice, the involvement of PC professionals facilitated access to resources and education that helped caregivers manage the patient’s illness [56]. Psychologically, family caregivers perceive healthcare professionals as a crucial support network that helps lessen the weight of their responsibilities and diminish their feelings of loneliness [51]. Unaided caregivers who provided care at home without assistance from healthcare providers frequently expressed feelings of being overwhelmed and encountered difficulties, such as misinterpreting the symptoms of the patients [52].

In this current study, caregivers emphasized the significance of compassionate and close relationships in establishing trust with PC professionals. This reassured them that they were valued members of a collaborative team focused on enhancing the patient’s overall health and well-being [56]. Establishing a rapport involves dedicating time to patients, engaging in constructive encounters, and building trust through a combination of hope and honesty [57].

Previous evidence reinforces our findings, highlighting the advantages of home-based PC interventions for patients and caregivers. These interventions have positive effects on multiple aspects, including answering information needs, enhancing quality of life, decreasing hospital admissions, reducing patients’ symptom burdens and resource utilization, increasing family satisfaction rates, and increasing the likelihood of patients achieving home deaths [19,54,58,59,60].

In home-based PC, it is common to observe a conflict between the dedication of family caregivers to the patient and their perception of the hardship associated with caregiving [60,61]. However, our participants expressed a strong desire to actively participate in decision-making and provide support to patients by advocating for them and respecting their autonomy [62,63,64]. Caregivers’ awareness and their trust that health care professionals are watching over them may reduce feelings of being isolated and foster caregivers’ preparedness for loss and bereavement [65,66]. Peer support can also help to reduce isolation and loneliness by decreasing caregiver burden and improving the carer’s quality of life and bereavement adjustment [67]. It should be noted that inadequate readiness for death was linked to negative outcomes in the bereavement process, including complicated grief, anxiety, and sadness after loss [68]. Death unpreparedness may be seen as a risk factor for caregivers of patients with life-limiting illnesses [66,69].

Our study indicates that healthcare personnel possess a satisfactory level of awareness regarding the information and practical support required by family caregivers, particularly during the final phase. Furthermore, telehealth can ensure continuity of care [70] and enhance the bond between caregivers and healthcare practitioners [71]. Our findings demonstrate that the utilization of telehealth enhanced caregivers’ ability to connect with healthcare professionals while still being able to remain in the comfort of their own homes. Telehealth facilitates a patient’s desire to be at home in EoL and provides caregivers with access to professional guidance and support [72]. In parallel, monitoring symptoms at home enables the transmission of symptom information to HPCTs in order to prevent or manage pain and other symptoms [73]. Person-based factors, rather than organization constraints, were more commonly identified as obstacles in the delivery of PC via telehealth. This finding aligns with evidence in the prior literature [74,75], particularly, research emphasizing the significance of digital literacy for successfully delivering telemedicine care [76]. The findings also suggest that design flaws in digital advising systems have adverse effects on the usability and user-friendliness of certain caregivers, exacerbating their reliance on assistance [72].

Although telehealth allows for close interaction, the absence of in-person visits makes it challenging to evaluate social needs that extend beyond conventional health-related concerns such as pain management. However, the ability to see a patient’s surroundings and intervene can greatly enhance their overall quality of life. An effective way of facilitating this comprehensive approach is to provide hybrid care, where telehealth is incorporated into patient care but is not the only method of service delivery. The inclusion of telehealth as an additional option to traditional in-person care aligns with the findings of our study. Telehealth has the potential to enhance early integrated PC by offering greater accessibility to patients in their own homes, provided that the equipment and connectivity are reliable and urgent needs are evaluated and resolved during in-person visits [72].

### 4.1. Strengths and Limitations

The implementation of an interview study facilitated the comprehension of the home-based care experience delivered by family members. The coding was performed by the first author (P.C.). This could have introduced a certain degree of bias, which is a potential constraint. To mitigate any potential bias, the research team pursued a rigorous methodology involving an iterative process, namely discussing the findings. Therefore, any assumptions or preexisting ideas concerning informal home care were identified and discussed. The decision to exclusively recruit participants from one HPCT was adopted for practical reasons. However, this choice imposed limits on this study’s transferability. Nonetheless, we posit that the concerns expressed by participants may resemble those encountered in other regions of the country.

This was a purposive small study, only focused on qualitative analysis. The recruitment of family carers was challenging because of the time limitations given their many responsibilities. Further mixed method research use of large sample sizes is needed to better understand caregiving preparedness and telehealth use, as well as outcome measures for caregivers (i.e., burden, well-being, quality of life, perceived health, and digital literacy). Participating caregivers were mostly female and well-educated. All were white and spoke Portuguese fluently. Thus, the perspectives presented in this study may not reflect the experiences of caregivers with more diverse backgrounds. Future research should include additional sociodemographic variables in the sample selection process, such as income, employment type, dependency levels of care recipients, and the extent of formal and informal support. These factors can greatly impact a carer’s capacity to deliver care. Additionally, future studies should triangulate the perspectives of caregivers, patients, and professionals to provide a comprehensive view of preparedness for caregiving and death. It might also be valuable to explore how telehealth can enhance training strategies to improve the psychological well-being of caregivers and provide them with better support.

Lastly, given the potential bias in the interpretation of the findings, the predominantly positive framing, and the research team’s experience in PC, the researchers strived to mitigate biases and uphold impartiality throughout the study. For instance, during data collection, we employed accurate phrasing of our questions, built rapport with each participant, and ensured that data collection was concurrent with data analysis. Furthermore, researcher reflexivity and consensus coding ensured unbiased, reliable, and accurate data analysis.

### 4.2. Implications for Practice

Considering the essential role that caregivers play in delivering care at home, it is necessary to ensure a strong level of engagement and assistance for caregivers in PC. In this vein, the provision of professional PC services in the home, along with a robust support system for informal caregivers, is invaluable. The support system could be enhanced by collaborating with healthcare experts to implement the new public health approach to PC, known as compassionate communities [77]. Additional measures are required to enhance EoL care, specifically focusing on home-based family care, particularly in nations with limited resources.

This study also highlights the broad influence of family dynamics on decision-making process in PC and how the bond between the patient and family caregiver shapes their preferences. Healthcare professionals need to consider how the extended family influences the level of agreement or disagreement between patients and family caregivers, as well as the expectations of both parties regarding their roles within the family. Engaging in discussions about future care via advanced care planning can enable patients and family caregivers to adapt to changes in their care preferences [78].

Furthermore, enabling healthcare professionals to enhance and strengthen their expertise in PC—for example, using care navigators—would facilitate the provision of timely and comprehensive supportive care. In this sense, telehealth services should be considered to achieve the full potential of PC [79]. Telehealth, the practice of delivering virtual healthcare services using Internet platforms, is widely acknowledged as valuable, particularly for providing after-hours telephone assistance and facilitating video conferences for interactive case discussions [80]. The availability of online caregiving resources is contingent upon Internet connectivity. Consequently, in areas where certain groups have limited access to the Internet, a telehealth system that offers both online and offline services for family caregivers could prove beneficial. Further research should also focus on investigating the telehealth experiences among individuals with life-limiting illnesses and the oldest-old caregivers. It is crucial to examine the varying experiences of these individuals in terms of usability and knowledge related to telehealth. If family caregivers are provided with training and educational interventions, telehealth modalities can be effectively employed to deliver a diverse array of skills.

## 5. Conclusions

As far as we know, this is one of the first studies to investigate informal home-based PC in Portugal from the viewpoint of family caregivers. Three main themes emerged: (1) becoming a caregiver, (2) support-from-home palliative care team, and (3) telehealth in palliative home care. This study emphasizes the substantial advantages of home-based PC interventions, enhancing the caregiver’s satisfaction rates and raising the probability of patients reaching death at home. Furthermore, they reduce the frequency of healthcare use, particularly hospital admissions and trips to the emergency department. Assistance by professionals and health services should be improved by developing a higher level of awareness and giving priority to the needs and readiness of carers, who have a unique position as both providers and recipients of care. Services and healthcare providers should improve their communication strategies, actively promote meaningful inclusion, tackle access difficulties, and enhance the support provided in their respective roles. This highlights the importance of a person-centered approach in EoL care and strengthens the long-term sustainability of the healthcare system through the valorization of informal care.

## Figures and Tables

**Table 1:** Six-phase process of reflexive thematic analysis [46,47].

Analytic Phases	Description
**Phase one: familiarization with the data**	Examining and reviewing the complete dataset to gain a deep understanding of the collected information.
**Phase two: generating initial codes**	Coding is the systematic procedure used to generate concise descriptive or interpretive labels for components of information that may be pertinent to the research question(s).
**Phase three: generating themes**	This phase commences once all pertinent data items have been encoded. The emphasis transitions from the analysis of individual data elements within the dataset to the analysis of the collective significance and relevance throughout the dataset.
**Phase four: reviewing potential themes**	Systematic examination of the candidate themes connected with the coded data items and the complete dataset.
**Phase five: defining and naming theme**	During this stage, the researcher produces a comprehensive examination of the thematic framework. Each distinct topic and sub-theme must be articulated with both the dataset and the research question(s).
**Phase six: producing the report**	Providing a succinct and captivating narrative of the data, both within and across themes.

**Table 2:** Participants’ background (n = 13).

Participant	Sex	Age	Illness Trajectory (Care Recipient)	Education Level	Relationship with Care Recipient	Time in the Caregiver Role
P1	Male	63	Heart failure	9th grade (3rd cycle of basic education)	Son	1.5 years
P2	Female	44	Cancer	Licentiate’s degree	Granddaughter	1 year
P3	Female	57	Cancer	12th grade (secondary education)	Daughter	8 months
P4	Female	53	Cancer	Licentiate’s degree	Daughter	1 year
P5	Female	66	Cancer	Licentiate’s degree	Daughter	7 years
P6	Male	70	Cancer	4th grade (1st cycle of basic education)	Husband	4 months
P7	Male	48	Neurodegenerative disease	12th grade (secondary education)	Husband	2.5 years
P8	Female	62	Dementia	Licentiate’s degree	Daughter	15 years
P9	Female	82	Dementia	4th grade (1st cycle of basic education)	Wife	4 years
P10	Female	56	Dementia	9th grade (3rd cycle of basic education)	Daughter	3.5 years
P11	Female	53	Cancer	12th grade (secondary education)	Wife	5 months
P12	Female	60	Cancer	4th grade (1st cycle of basic education)	Daughter	2 years
P13	Female	60	Cancer	4th grade (1st cycle of basic education)	Wife	5 years

**Table 3:** Main themes and subthemes.

Main Themes	Sub-Themes
Become a caregiver	Caregiving as a natural decision
	Imposed caregiving
Support from home palliative care team	Compassionate caring relationships
	Managing complex situations and emotional turmoil
	Participating in treatment decision-making
Telehealth in palliative home care	Symptom management and self-care promotion
	Drawbacks and challenges of telehealth

## Data Availability

All data generated or analyzed during this study are included in this article. This article is based on the first author’s master’s dissertation in Palliative Care at the School of Health Sciences—Polytechnic University of Leiria.

## Supplementary Materials

The following supporting information can be downloaded at: https://www.mdpi.com/article/10.3390/healthcare12191915/s1, Table S1: Consolidated criteria for reporting qualitative studies (COREQ): 32-item checklist.
